# Unveiling the Potent Fibrino(geno)lytic, Anticoagulant, and Antithrombotic Effects of Papain, a Cysteine Protease from *Carica papaya* Latex Using κ-Carrageenan Rat Tail Thrombosis Model

**DOI:** 10.3390/ijms242316770

**Published:** 2023-11-26

**Authors:** Hye Ryeon Yang, Most Nusrat Zahan, Yewon Yoon, Kyuri Kim, Du Hyeon Hwang, Woo Hyun Kim, Il Rae Rho, Euikyung Kim, Changkeun Kang

**Affiliations:** 1 Department of Basic Veterinary Medicine, College of Veterinary Medicine, Gyeongsang National University, Jinju 52828, Republic of Korea; tkwk565@naver.com (H.R.Y.); zahan.nusrat.gnu@gmail.com (M.N.Z.); puruuum11@gmail.com (Y.Y.); okhea4me@gmail.com (K.K.); pooh9922@hanmail.net (D.H.H.); woohyun.kim@gnu.ac.kr (W.H.K.); ekim@gnu.ac.kr (E.K.); 2 Institute of Animal Medicine, Gyeongsang National University, Jinju 52828, Republic of Korea; 3 Institutes of Agriculture and Life Science, Gyeongsang National University, Jinju 52828, Republic of Korea; irno12@gnu.ac.kr

**Keywords:** papain, cysteine protease, blood coagulation, thrombosis, κ-carrageenan

## Abstract

While fibrinolytic enzymes and thrombolytic agents offer assistance in treating cardiovascular diseases, the existing options are associated with a range of adverse effects. In our previous research, we successfully identified ficin, a naturally occurring cysteine protease that possesses unique fibrin and fibrinogenolytic enzymes, making it suitable for both preventing and treating cardiovascular disorders linked to thrombosis. Papain is a prominent cysteine protease derived from the latex of *Carica papaya*. The potential role of papain in preventing fibrino(geno)lytic, anticoagulant, and antithrombotic activities has not yet been investigated. Therefore, we examined how papain influences fibrinogen and the process of blood coagulation. Papain is highly stable at pH 4–11 and 37–60 °C via azocasein assay. In addition, SDS gel separation electrophoresis, zymography, and fibrin plate assays were used to determine fibrinogen and fibrinolysis activity. Papain has a molecular weight of around 37 kDa, and is highly effective in degrading fibrin, with a molecular weight of over 75 kDa. Furthermore, papain-based hemostatic performance was confirmed in blood coagulation tests, a blood clot lysis assay, and a κ-carrageenan rat tail thrombosis model, highlighting its strong efficacy in blood coagulation. Papain shows dose-dependent blood clot lysis activity, cleaves fibrinogen chains of Aα, Bβ, and γ-bands, and significantly extends prothrombin time (PT) and activated partial thromboplastin time (aPTT). Moreover, the mean length of the infarcted regions in the tails of Sprague–Dawley rats with κ-carrageenan was shorter in rats administered 10 U/kg of papain than in streptokinase-treated rats. Thus, papain, a cysteine protease, has distinct fibrin and fibrinogenolytic properties, suggesting its potential for preventing or treating cardiovascular issues and thrombosis-related diseases.

## 1. Introduction

Papain (EC 3.4.22.2), a potent proteolytic enzyme, has attracted significant interest from both researchers and industries for its remarkable protein-cleaving abilities. Papain is sourced from the latex of the *Carica papaya*, a tropical fruit-bearing plant native to Central and South America [1]. Throughout ancient times, various parts of the papaya plant, including fruit, leaf, seed, bark, and latex, have been utilized for their therapeutic applications [2]. Latex is a complex mixture of chemical compounds exhibiting diverse chemical activities. These compounds are believed to play a collective role in the plant’s defense system [3]. Papaya latex contains a significant concentration of papain enzyme, which has the ability to break down proteins into smaller peptides and amino acids by hydrolyzing the peptide bonds that hold them together [4]. Its proteolytic activity, substrate specificity, and stability under diverse conditions make it a versatile tool in fields ranging from food to pharmaceuticals and biotechnology [5]. Papain’s therapeutic potential is evident in the pharmaceutical sector, where it is studied for its anti-inflammatory and wound-healing properties [6].

Presently, significant efforts are being directed towards discovering natural products that can serve as effective supplements or potential alternatives to the currently used antithrombotic drugs [7,8,9]. When blood vessels suffer an injury, platelets become activated upon encountering the exposed subcutaneous matrix. They then gather and aggregate at the site of the injury, effectively halting the bleeding process [10]. Platelet aggregation occurs due to the rupture of atherosclerotic plaques or endothelial injury, which can result in acute thrombotic occlusive ischemic events when the blood supply to tissues is inadequate or gets disrupted due to the presence of a thrombus [11]. Blood coagulation and platelet-mediated primary hemostasis play critical roles in protecting against bleeding [12]. The coagulation system responds to endothelial rupture, leading to platelet plug formation [13]. This triggers a coordinated response leading to the formation of a platelet plug, which initially occludes the vascular lesion. Anticoagulant mechanisms play a vital role in carefully controlling coagulation, prevailing over procoagulant forces in normal conditions [14]. However, imbalances between the procoagulant and anticoagulant systems, whether due to genetic or acquired factors, can lead to bleeding or thrombotic disorders [15].

Over the past years, significant research and development efforts have been dedicated to antithrombotic drugs, which can be categorized into three main groups: anticoagulation, antiplatelet aggregation, and fibrinolysis [16]. These drug classes hold promising potential as therapeutic approaches for addressing arterial and venous thrombosis [17,18]. Heparin, warfarin, and their derivatives are, in clinical settings, primarily used to inhibit blood coagulation factors [19]. Additionally, numerous antiplatelet drugs like aspirin, clopidogrel, and abciximab are extensively used to reduce the risk of cardiovascular diseases [20,21]. Moreover, fibrinolytic agents, such as streptokinase, tissue plasminogen activator (t-PA), and reteplase, are utilized to promote the dissolution and removal of formed blood clots [22,23].

However, the present fibrinolytic enzymes and thrombolytic drugs in clinical settings have a number of adverse effects, including bleeding problems and hemorrhage [24]. As a result, many researchers are looking for therapeutic alternatives from natural sources for the treatment of thrombotic diseases since they are composed from multiple constituents, each with the potential to target multiple sites [7]. In traditional medicine, various cysteine protease preparations have been studied to enhance platelet aggregation and address thrombosis-related diseases [25]. Despite the potential therapeutic benefits of cysteine proteases for cardiovascular illnesses, there is a lack of significant human and animal trials to thoroughly investigate these effects. Hence, the objective of this study is to investigate the fibrino(geno)lytic, anticoagulant and antithrombotic characteristics of papain, a cysteine protease derived from unripe fruit through both in vitro and in vivo experimental procedures.

## 2. Results

### 2.1. SDS-PAGE Profile of Papain

**Figure 1:**
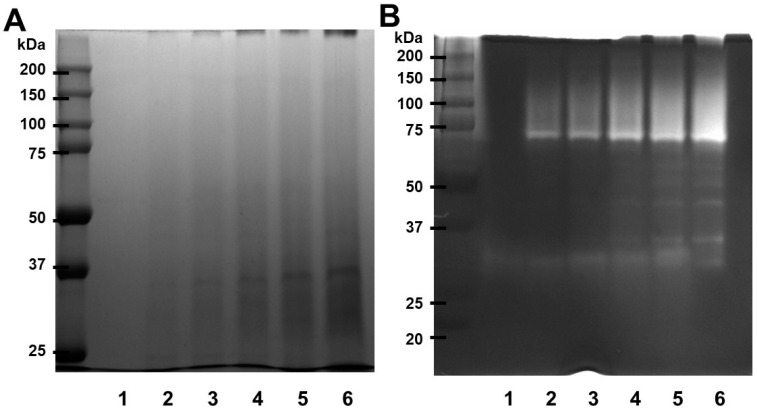
SDS-PAGE profile and fibrinolytic activity of papain. (**A**) Papain was submitted to SDS electrophoresis under non-reducing conditions. The gels were treated with a 0.125% solution of Coomassie blue stain. (**B**) Different concentrations of papain were used for fibrin zymography. The presence of clear zones in the fibrin gel indicated the areas where proteolytic activity occurred. Lane 1—0, lane 2—0.125, lane 3—0.25, lane 4—0.5, land 5—1, lane 6—2 (U/mL).

The electrophoretograms of the SDS-PAGE gel produced under decreasing conditions are shown in Figure 1A. Papain has a molecular weight of about 37 kDa, with a similar result from Nurhayati et al. [26]. Fibrin zymography was conducted with different papain concentrations to investigate the fibrinolytic activity of papain. Papain has a molecular weight exceeding 75 kDa, and its fibrinolytic activity, as illustrated in Figure 1B, displayed its efficacy in degrading fibrin. The results suggest that papain has the ability to break down fibrin, indicating its potential in its capacity as a fibrinolytic enzyme.

### 2.2. Effect of Temperature and pH on Protease Activity and Stability

**Figure 2:**
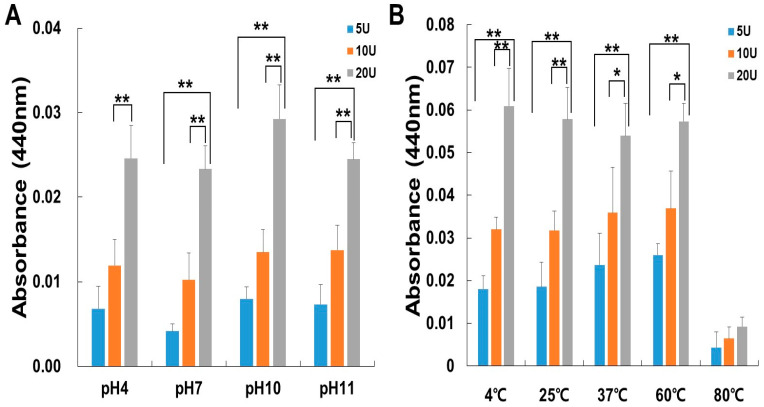
The influence of pH (**A**) and temperature (**B**) on the enzyme activity of papain. The enzyme activity was assessed using azocasein assays, and the measurements were taken at 440 nm. (**A**) The papain activity was examined by incubating at 37 °C for 30 min while varying the pH levels at 4, 7, 10, and 11. (**B**) Papain activity was evaluated following incubation over a temperature range spanning from 4 °C to 80 °C. The data shown are the mean ± SD (*n* = 4) of three independent experiments. * *p* < 0.05, and ** *p*< 0.01 vs. each group after ANOVA and Dunnett’s test.

The activity of papain was tested at different temperatures to determine the optimum temperature for its activity. The thermostability of papain was carried out by pre-incubating the enzyme for 60 min at a temperature range of 4 to 80 °C, as shown in Figure 2A. Papain was stable up to 60 °C, followed by a decrease in activity at 80 °C. When papain was pre-incubated at a higher temperature (80 °C) for 60 min, a notable decline in its activity was observed. Moreover, the thermostability of all groups was increased in a dose-dependent manner. Papain was tested in a variety of buffer systems with various pH values (see Materials and Methods) to find the ideal pH for papain activity. All pH was optimum for papain use (Figure 2B). The activity steadily increased dose-dependently. As the temperature rose, the activity of papain increased, reaching its peak at 60 °C, and remained stable across all pH levels. Papain is a well-documented proteolytic enzyme with established stability parameters. Previous research has indicated Papain exhibited its peak enzymatic activity across a broad pH spectrum, ranging from 5.8 to 7.0, and a wide temperature range, specifically between (50–57 °C), with casein as the substrate [27,28]. Also, papain exhibits its highest level of activity within the pH range of 4.0 to 6.0, even though it retains its enzymatic activity over a wide pH spectrum [29]. Our experimental findings indicate that papain’s stability is not confined to the previously established conditions but extends to a pH as high as 11. This also offers exciting prospects for its utilization in a broader range of applications, while prompting a reevaluation of the enzyme’s stability parameters in future research.

### 2.3. Fibrinolytic Activity of Papain

**Figure 3:**
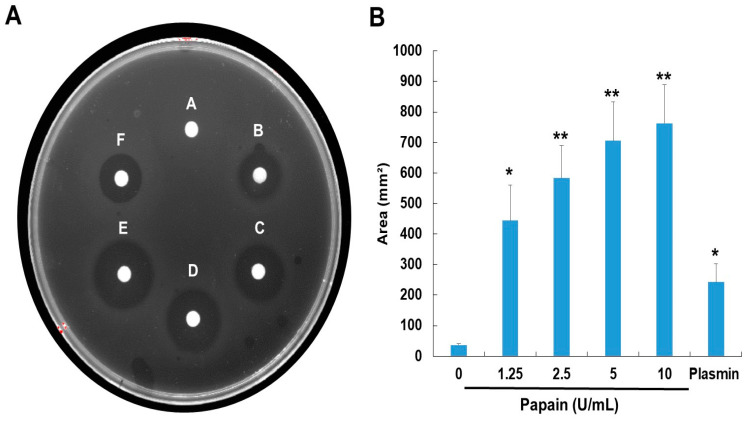
Fibrinolytic activity of papain. (**A**) Fibrinolytic activity was assessed using a fibrin plate assay. The samples were applied to the disc on the plate and then incubated at 37 °C overnight. (**B**) Fibrinolytic activity was quantified based on the size of clear zones. A—0, B—0.0125, C—0.025, D—0.05, E—0.1 (U), F—plasmin 2 U/mL. The data shown are the mean ± SD (*n* = 4) of three independent experiments. * *p* < 0.05, and ** *p* < 0.01 vs. control group after ANOVA and Dunnett’s test.

The papain was allowed to react on fibrin plates to ascertain if it had a direct or indirect fibrinolytic action (Figure 3). Papain’s fibrinolytic activity was greater than that of plasmin (positive control), and it produced the creation of a clear zone in a dose-dependent manner. After 24 h of incubation, papain’s fibrinolytic activity was detected, and from that point on, an obvious hollow began to appear progressively. Papain’s fibrinolytic action was thus proven.

### 2.4. Fibrinogenolytic Activity of Papain

**Figure 4:**
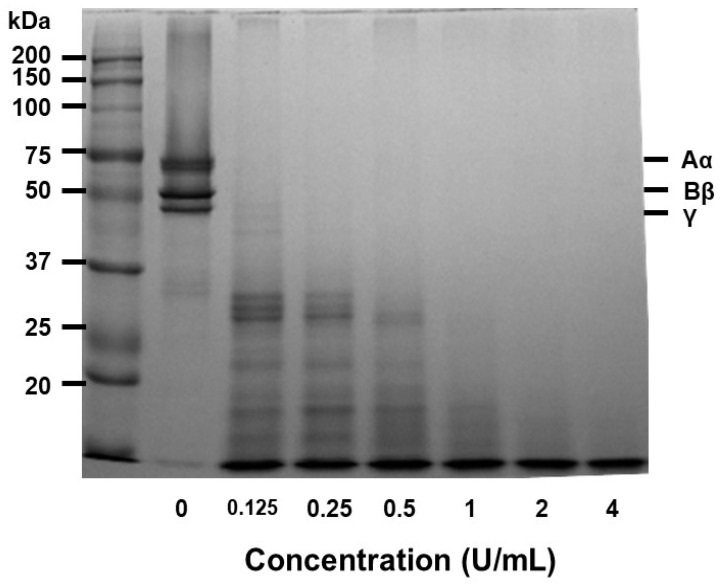
The impact of papain on fibrinogen. Fibrinogenolytic activity was assessed using SDS-PAGE after incubating human fibrinogen with different concentrations of papain at 37 °C for 30 min. The mixture samples were subjected to electrophoresis on a 7.5% SDS-PAGE gel and subsequently stained with Coomassie blue. Fibrinogen is composed of three polypeptide chains: Aα, Bβ, and γ.

Papain was incubated with human fibrinogen at 37 °C to examine how it was able to hydrolyze it, and the reaction was run on a 7.5% SDS-PAGE. To evaluate the enzyme’s capacity to hydrolyze fibrinogen, the reaction product was tested on a 7.5% SDS-PAGE. Papain readily cleaved fibrinogen’s Aα and Bβ chains in a dose-dependent manner (Figure 4). The fibrinogen, Aα, Bβ, and γ-chain were promptly broken down in 0.05 U/mL of papain.

### 2.5. Blood Clot Lysis

**Figure 5:**
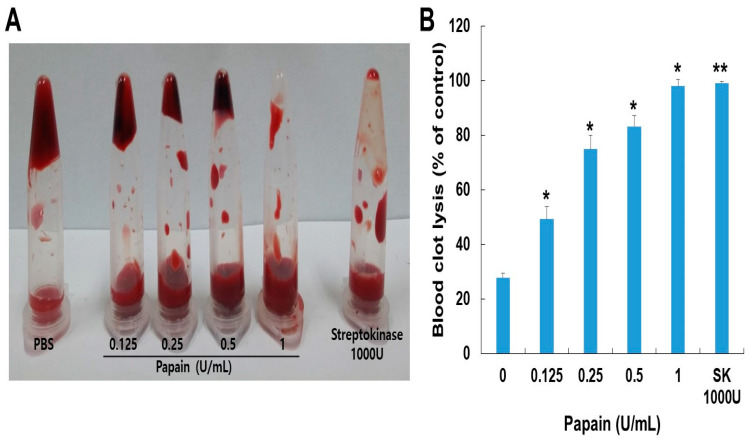
(**A**) The impact of papain on in vitro thrombolysis. (**B**) Clot lysis was examined through different concentrations of papain alongside streptokinase for 24 h. Streptokinase served as the positive control. The data shown are the mean ± SD (*n* = 4) of three independent experiments. * *p* < 0.05, and ** *p* < 0.01 vs. control group after ANOVA and Dunnett’s test.

A dog blood clot lysis assay was conducted to assess the impact of papain on blood clots. As shown in (Figure 5), papain showed clot lysis activity in a dose-dependent manner. Papain demonstrated a significantly higher clot lysis ability compared to the PBS-treated group (negative control) and outperformed the streptokinase-treated group (positive control). The size of the clot was significantly reduced by papain, particularly when using a concentration of 0.1 U/mL, which resulted in the dissolution of the blood clots in 98.6% of the cases.

### 2.6. Anti-Coagulation Effect of Papain

Dog blood was used to assess the aPTT and PT levels to examine the impact of papain on anticoagulant properties. In comparison to individuals who were not treated with papain, those who received treatment saw an improvement in their PT and aPTT values (Table 1). The PT and aPTT were extended over 35 s and 200 s at the indicated papain concentration (0.8 U/mL). As a consequence, papain has components acting as anticoagulants via the regulation of the coagulant pathways; thus, papain acts as an anticoagulant.

### 2.7. κ-Carrageenan-Induced Rat Tail Thrombosis Model 

**Figure 6:**
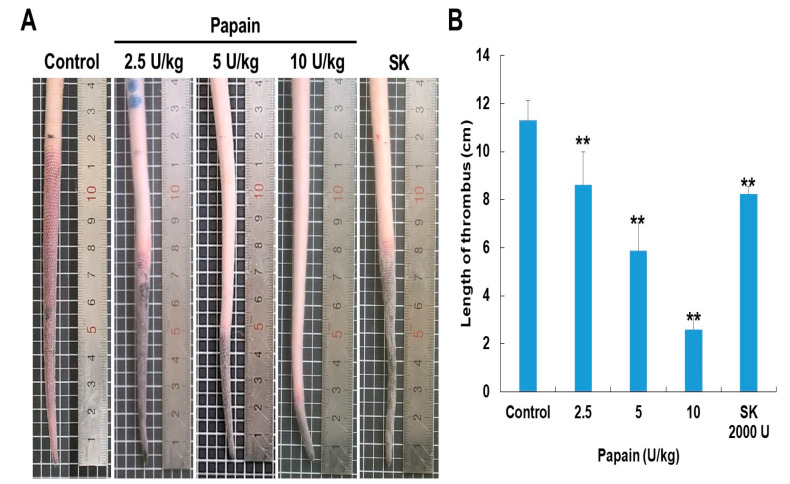
Papain prevents κ-carrageenan-induced thrombosis in Sprague–Dawley rats. (**A**) Representative photographs taken 48 h after carrageenan injection. In the control group (κ-carrageenan injected alone), the thrombus length measured 11.6 ± 0.4 cm. The thrombus length in the papain-treated groups showed a reduction in a dose-dependent manner. (**B**) The bar diagram illustrates the inhibitory effects of papain and streptokinase on tail thrombus formation at 48 h. Data are expressed as the mean ± SD (*n* = 5) of three independent experiments. ** *p* < 0.01 vs. control group after ANOVA and Dunnett’s test.

The thrombolytic activity of papain was examined using a rat tail thrombosis model induced by κ-carrageenan. Following κ-carrageenan administration via the rat tail route, the tails of the rats exhibited an auburn color, indicating the formation of thrombi (Figure 6). The control group, which received κ-carrageenan alone, displayed an average thrombus length of 11.6 ± 0.4 cm. In this group, the thrombus in the tail tip transitioned from a wine color to auburn and subsequently advanced to significant necrosis within 48 h following the injection of κ-carrageenan. But papain effectively lessened these effects. The papain-treated group (10 U/kg) exhibited a mean thrombus length of 2.8 ± 0.4 cm. The study utilized streptokinase as the positive control in the experiment. The average length of the thrombus in the streptokinase-treated group was 8.7 ± 0.2 cm.

## 3. Discussion

This study investigated the potential of papain in preventing fibrinolytic, anticoagulant, and antithrombotic activities. We explored papain’s impact on fibrinogen and blood coagulation, highlighting its efficacy in degrading fibrin with a molecular weight over 75 kDa. Hemostatic performance was validated through blood coagulation tests and a rat tail thrombosis model, demonstrating papain’s strong effectiveness. The enzyme exhibited dose-dependent blood clot lysis, cleaved fibrinogen chains, and significantly prolonged prothrombin time and activated partial thromboplastin time. The study suggests that papain, a cysteine protease, possesses distinct fibrin and fibrinogenolytic properties with potential applications in preventing or treating cardiovascular issues and thrombosis-related diseases.

With the rapid advancements in science and technology, traditional medicine has emerged as an appealing subject for researchers [30]. Particularly, there is a continuous interest in conducting evidence-based research on natural products such as papain, not only to explore their traditional uses but also to potentially develop them into formal medications [31]. Papain, derived and identified from *C. papaya*, is categorized as a cysteine protease and recognized as a proteolytic enzyme, relying on the presence of a free sulfhydryl group for its enzymatic activity [32]. The plant’s medicinal properties can be attributed to its antioxidative nature, which offers cellular protection against oxidative stress-induced harm [33]. Papain is an extensively utilized proteolytic enzyme, renowned for its contributions to meat tenderization and aiding digestion [34]. Papain is also regarded as having significant pharmacological potential, with drug-like properties that might aid in the treatment of atherosclerosis and related illnesses. However, the early discovery of papain’s influence on the transition from fibrinogen to fibrin shed light on the fibrinolytic properties of papain, providing a fundamental comprehension of this enzyme’s involvement in the coagulation process [35].

Our experimental utilization of papain as a cysteine protease demonstrated remarkable efficacy in improving hemostasis, particularly in heparin-treated scenarios, providing a cost-effective and promising solution for surgical bleeding control [36]. Its effectiveness lies in addressing monocyte-platelet aggregate (MPA)-regulated inflammation [37]. Thrombotic disorders, marked by the development of blood clots, play a substantial role in causing illness and death on a global scale [37]. Hence, the investigation of natural compounds possessing potential thrombolytic properties holds immense promise in the pursuit of alternative and efficient therapeutic approaches [38]. In this study, we investigated the fibrino(geno)lytic, anticoagulant, and antithrombotic activities of papain, a cysteine protease derived from papaya plant latex, using the κ-carrageenan-induced rat tail thrombosis model.

Our findings demonstrated that the combination of SDS-PAGE and fibrin zymography upyielded comprehensive insights into the structural stability and fibrinolytic potential of papain (Figure 1). In comparison to our prior research with ficin (24–50 kDa) and the current study using papain (37–75 kDa), they are fibrinolytic enzymes capable of digesting fibrin. When compared with our previous paper about pH stability and thermostability, papain shows broader specificity [25]. Ficin’s optimal pH for activity was determined to be 7 in a phosphate buffer, with a notable decline in activity observed at pH 10. Additionally, its stability peaked at 60 °C when exposed to pH 7, indicating a pH and temperature-dependent nature to ficin’s performance; papain’s activity exhibited a dose-dependent increase with rising temperatures, culminating at 60 °C, and it displayed stability across various pH levels. The two exhibit differences in molecular weight as detected under different conditions, indicating potential conformational changes or interactions. These findings not only enhance our understanding of papain’s activity but also pave the way for its potential application in medical and therapeutic contexts [34]. Additionally, the efficacy and safety of papain-based treatments may vary, and further research is needed to establish their clinical effectiveness.

The fibrin plate assay results also demonstrate papain’s potential as a fibrinolytic agent, with clear zones rising in size as concentrations increase (Figure 2). Papain showed a broader range of fibrinolytic activity (0 U/mL to 10 U/mL) compared to ficin (0 U/mL to 0.1 U/mL) [25]. Particularly at elevated concentrations, it appears to approximate the effectiveness displayed by plasmin (Figure 3). This emphasizes papain’s effectiveness in fibrin degradation, which is crucial for clot removal and tissue remodeling. These findings are pertinent to medical contexts where fibrinolysis is crucial in conditions like wound healing, thrombosis, and heart disorders [39,40,41]. Papain also exhibited a significant fibrinogenolytic property, with the Aα and Bβ subunits being susceptible to degradation, while the γ subunits were completely hydrolyzed with an increasing dose (Figure 4).

Fibrinolysis is a critical process involved in dissolving blood clots by cleaving fibrin, the main component of thrombi [42]. Papain demonstrated a significant influence on blood clot lysis across the range of concentrations through in vitro analysis. Incubating the whole blood clot with papain (0.0125–0.1 U) resulted in the dissolution of 99% of the clot, comparable to the effect achieved via streptokinase (1000 U/mL). The observed clot lysis potential in response to papain treatment, compared to streptokinase, suggests papain’s potential as a thrombolytic agent (Figure 5). 

Papain was evaluated via in vitro study using PT and PTT assays, which can be used to study the extrinsic and intrinsic coagulation cascade. PT assays primarily involve procedures that initiate the extrinsic coagulation factor (factor VII) and common (factors II, V, and X) pathways, as well as fibrinogen [43], whereas the aPTT assay involves procedures that initiate intrinsic coagulation system, comprising factors VIII, IX, XI, and XII [44]. In this study, we found that papain is able to prolong PT and aPTT even at a concentration of 8 U/mL Prolongation of PT can be due to a deficiency in any of the factors involved in the extrinsic and common pathways of blood coagulation or the presence of papain. A possibility for explaining PT prolongation is the changing of factors by papain, which needs to be verified. The prolongation of aPTT indicates the inhibition of thrombus formation or the polymerization of the fibrin that has formed. Therefore, the results obtained in this study suggest that papain could also have anticoagulant activity (Table 1).

Fibrinolytic enzymes have been successfully isolated from a wide range of sources [45]. Streptokinase, urokinase, pro-urokinase, reteplase, and alteplase, among other fibrinolytic enzymes and thrombolytic medicines presently utilized in clinical settings, have considerable undesirable physiological side effects. These include a heightened bleeding risk, a short plasma half-life, restricted fibrin specificity, and the necessity for high therapeutic dosages [23,46]. As a consequence, the search for more inexpensive and efficient fibrinolytic enzymes and thrombolytic medications becomes a crucial issue. Numerous studies have revealed the utilization of papain in traditional medicine [34]. The results of the present study indicate that papain holds promise as a potential source of fibrinolytic and thrombolytic agents. 

Upon intravenous administration, κ-carrageenan induces the production of histamine and serotonin by immune cells, leading to acute inflammation of blood vessels in various animal species [47]. So, κ-carrageenan administration in the tail veins is strongly linked to thrombus development and inflammation. We therefore used the rat-tail thrombus model induced by κ-carrageenan to evaluate the in vivo antithrombotic activity of papain. Papain has a dose-dependent antithrombotic potential in rat tails, either delaying the development of thrombi or dissolving pre-existing ones (Figure 6). In our previous study, we also investigated the effectiveness of ficin in both preventing thrombus formation and dissolving pre-formed thrombi [25]. Our findings suggest that papain (10 U/kg, a mean thrombus length of 2.8 ± 0.4 cm) exhibits superior thrombus-modulating properties compared to ficin (10 U/kg, a mean thrombus length of 8.8 ± 0.3 cm), presenting it as a potential candidate for future therapeutic interventions targeting thrombotic disorders. Therefore, papain proves to be a highly effective antithrombotic agent, holding significant potential for further exploitation and development. This initial examination of the effects of papain on the coagulation and fibrinolysis systems carries broad implications across various indications. The research primarily identifies cysteine proteases that offer promise as possible sources for pharmacological therapies for various hemostatic disorders. There is increasing evidence that cysteine proteases are linked to the physiological processing of chemokine [48]. One example is cathepsins, which were originally isolated from macrophages, and inflammatory cytokines increase cathepsin secretion from macrophages [49]. Furthermore, recent in vivo studies of cathepsin demonstrate that this enzyme is involved in leukocyte infiltration, endothelial cell invasion, and neovascularization [47]. Thus, cysteine protease may have a function in regulating cytokines and chemokines under inflammation, implying that papain could play a significant role in atherogenesis and thrombosis-related disorders.

## 4. Materials and Methods

### 4.1. Chemicals and Reagents

Papain (Catalog No. P4762), fibrinogen (Catalog No. F8630, type I-S from bovine plasma), and thrombin (Catalog No. T7326, from bovine plasma): Sigma-Aldrich (St. Louis, MO, USA) supplied the materials. Vetscan^®^VSpro (Abaxis Inc., Union City, CA, USA) provided the PT and aPTT combination test kits. All reagents utilized in this study were of the highest purity grade.

### 4.2. Sodium Dodecyl Sulfate-Polyacrylamide Gel Electrophoresis (SDS-PAGE)

Electrophoresis was performed following the Laemmli [50] method, employing a 12% separating gel and a 4% stacking gel. Papain samples were added to a non-reducing sample buffer composed of 4% SDS, 125 mM Tris-HCl (pH 6.8), 20% glycerol, and 0.01% bromophenol blue, which was stored at −20 °C until use. Using the Tris-glycine running buffer, papain was electrophoresed at 100 V for 90 min. Molecular weight markers ranging from 10 to 200 kDa (Precision plus Protein TM Standards, Bio-Rad, Hercules, CA, USA) were run in parallel to determine molecular weight. Following electrophoresis, the gel was stained in 40% methanol and 10% acetic acid with 0.125% Coomassie Blue.

### 4.3. Proteolytic Activity Assay

Proteolytic activity was evaluated as described by Segers et al. with some slight modifications [51]. A volume of 100 L of the reaction mixture contained papain, 150 µL of reaction sodium bicarbonate buffer (0.5% solution, pH 8.3) (Buffer A), and 250 µL of 2.5% azocasein (*w*/*v*) dissolved in Buffer A. After 30 min of operation at 37 °C, the assays were finished with 400 L of 10% (*w*/*v*) trichloroacetic acid. The precipitated protein was removed by centrifuging the reaction mixture for 20 min at 12,000 rpm. A UV/visible spectrophotometer (PowerWaveTMXS, BioTek Instruments, Inc., Winooski, VT, USA) was used to measure the absorbance at 440 nm after neutralizing the supernatant (500 µL) by adding 300 µL of sodium hydroxide (500 mM).

### 4.4. Effect of Temperature and pH on Protease Activity and Stability

An enzyme experiment for papain was used to determine the optimum temperature and pH by following Aissaoui, N., et al., which was slightly modified [52]. The papain was preincubated at temperatures ranging from 4 to 80 °C for 60 min to assess its thermal stability. The ideal pH was identified by doing tests at 4 °C in buffers with varied pH values (pH 4, acetate buffer; pH 7, phosphate buffer; pH 10.0 and 11.0, glycine-NaOH buffer). The papain was maintained at 4 °C for 30 min in various buffers. 

We used pH ranges from 4.0 to 11.0 to test for pH stability. As previously mentioned, residual proteolytic activity was calculated and represented as a percentage of the original activity, which was taken to be 100%.

### 4.5. Fibrin Plate Assay

The method for assessing fibrinolytic activity was conducted following Astrup and Mullertz’s procedure, with slight adjustments made to accommodate the experimental conditions [53]. Briefly, to create plasminogen-free plates, a solution was prepared by combining 5 mL of 0.6% *w*/*v* fibrinogen (Calbiochem, Darmstadt, Germany) with 1% agarose in 50-mM Tris-HCl buffer at (pH 7.8). To initiate the clotting process, 100 µL of thrombin (100 NIH U/mL, Sigma-Aldrich, St. Louis, MO, USA) was added to the mixture. The prepared plates were allowed to stand at room temperature (25 °C) for 30 min to facilitate the formation of a fibrin clot. Small dried filter paper disks with a diameter of 5 mm were placed on the fibrin layer and then incubated at 37 °C for 24 h. Following this, drops of either 20 μL papain at varying concentrations (0, 0.0125, 0.025, 0.05, or 0.1 U/mL) or a positive control (plasmin at 2 U/mL) were applied to the disks. Fibrin degradation was evident through the presence of clear, translucent zones surrounding the disks. The efficacy of this degradation was directly proportional to the diameter of these zones, indicating a stronger and more extensive fibrinolytic activity. The fibrinolysis area (in mm^2^) was quantified by measuring the diameters of the fine rings formed around each sample using ImageJ 1.53t software. By determining the average width of the hydrolyzed clear zone, the extent of lysis induced by each sample was calculated.

### 4.6. Examination of Fibrinolytic Activity Using Fibrin Zymography

The zymography assay assessed fibrinolytic activity by utilizing fibrin as the substrate. For the preparation of the zymography gel, a solution containing 20 mM sodium phosphate buffer at pH 7.4 was utilized. This solution included dissolved fibrinogen at a concentration of 0.6 mg/mL and thrombin at a concentration of 0.01 unit/mL. The copolymerization process involved mixing these components with 12% polyacrylamide to create the appropriate gel for the experiment. To analyze papain, a non-reducing sample buffer was subsequently employed; the prepared papain samples were subjected to gel electrophoresis at 100 V and 4 °C. After the electrophoresis process, the gel was subjected to washes two times, each lasting for 30 min, in a 2.5% Triton X-100 solution to remove SDS. Following the treatment with 20 mM Tris (pH 7.4), 0.5 mM calcium chloride, and 200 mM sodium chloride at 37 °C for 16 h, the gel was subsequently stained with 0.125% Coomassie blue. The regions of fibrinolytic activity were visually evident as clear zones on the gel.

### 4.7. Fibrinogenolytic Activity

The fibrinogenolytic activity of papain was investigated following the protocol described by Matsubara et al. [54]. In brief, the experiment involved adding reaction buffer (pH 7.4, 200 mM sodium chloride, 0.5 mM calcium chloride, and 20 mM Tris) to 15 µL of bovine fibrinogen at a concentration of 20 mg/mL. The mixture was then incubated at 37 °C for specific time periods and dosages. To analyze the digested products, a 7.5% SDS-PAGE gel was employed.

### 4.8. Blood Clot Lysis Assay

Venous blood samples were collected from healthy Mongolian dogs and dispensed into multiple pre-weighed, sterile microcentrifuge tubes, with each tube containing 500 μL of blood. The tubes were then incubated for 45 min at a temperature of 37 °C. Upon the formation of blood clots, all the serum was carefully removed from each tube, and the tubes containing the clots were reweighed to determine their weight (clot weight = weight of tube with clot—weight of empty tube). Each microcentrifuge tube containing the blood clot was appropriately labeled, and varying concentrations of papain (ranging from 0 to 1 U/mL) were then introduced into the respective tubes. For the negative control, phosphate-buffered saline (PBS) was added to each tube containing clots, while for the positive control, streptokinase (SK, 1000 U) was added to each tube with clots. All the tubes were subjected to a 24 h incubation at 37 °C, during which they were closely monitored for any signs of clot lysis. Following the incubation period, the fluids from each tube were collected. Subsequently, the tubes were reweighed to ascertain the weight difference after the breakdown of the blood clots. The proportion of clot lysis was calculated by comparing the weight of the tubes before and after the clot lysis process.

### 4.9. PT/aPTT Measurement

Prothrombin time (PT) and activated partial thromboplastin time (aPTT) measurements were employed to investigate the impact of papain on blood coagulation. For the coagulation tests, blood samples were drawn from Mongolian dogs and mixed with a solution containing 3.2% sodium citrate (1:9, *v*/*v*) along with different papain concentrations. The mixture was allowed to incubate for 10 min at room temperature. The mixtures, following the 10 min incubation, were evaluated using an aPTT and PT combination test kit (Vetscan^®^VSpro PT/aPTT combination test cartridge, Abaxis Inc., Union City, CA, USA).

### 4.10. Experimental Animals

Four-week-old male Sprague–Dawley (SD, 150–160 g of body weight) rats were obtained from Samtako Inc. (Osan, Korea) and housed in the lab of the Gyeongsang National University animal research facility. The rats were housed in standard cages under conventional conditions, which included a 12 h light/dark cycle and ad libitum access to conventional chow meal (8% protein, 5% fat, 4.5% fiber, 8% carbohydrate, 0.7% calcium, 1.2% phosphorus, and 14% moisture) and water. The environmental conditions maintained an ambient temperature of 23 ± 2 °C, with a relative humidity ranging from 35 to 60 percent. The animal study protocol used in this research was approved by the Institutional Animal Care and Use Committee of Gyeongsang National University, with the protocol number GNU-200122-R0003.

### 4.11. κ-Carrageenan-Induced Rat Tail Thrombosis Model

We first determined the toxic doses of papain in rats. In our pilot study, 2.5 to 10 U/kg of papain was not found to have an abnormal or toxic effect when administered to rats (data not shown). A total of 25 male SD (5-week-old) rats were randomly allocated into five groups (*n* = 5 per group); group 1, vehicle-treated group (Control); group 2, 3, and 4, 2.5 U/kg, 5 U/kg, and 10 U/kg papain with κ-carrageenan, respectively; group 5, a dose of streptokinase 2000 U (mL/kg) with κ-carrageenan (positive control). First, κ-carrageenan (1 mg/kg) was dissolved in saline and injected into the dorsal tail vein of the rat. Following the ligation of a site 12 cm from the tip of the rat tail, 1 mg/kg of κ -carrageenan was administered intravenously to measure thrombogenesis. After being ligated for 20 min, the ligation was removed. The experimental groups were given papain and streptokinase intravenously for 1 h before receiving κ-carrageenan. The frequency of infarction and the length of the infarcted region at the tail tip were recorded 48 h after κ-carrageenan injection.

### 4.12. Statistical Analysis

The results were obtained from a minimum of two or three independent experimental conditions and were expressed as the mean ± standard deviation (S.D.). Statistical analysis was determined by *t*-test and one-way analysis of variance (ANOVA), followed by Dunnett’s post hoc test using SPSS version 13.0. A *p*-value less than 0.05 was considered statistically significant.

## 5. Conclusions

We investigated papain, a cysteine protease, for its varied fibrino(geno)lytic activities, anticoagulant, and antithrombotic effects using the κ-carrageenan-induced rat tail thrombosis model. The findings of the research provide valuable insights into the effects of papain on blood clotting and thrombosis formation. In addition, papain’s anticoagulant activity indicates its potential to reduce blood clot formation by inhibiting the coagulation cascade. These findings could lead the way for novel antithrombotic treatments, utilizing papain or its derivatives. Additional research is needed to explore papain’s impact on anti-inflammatory processes and uncover its molecular mechanism of action.

## Figures and Tables

**Table 1:** The anticoagulation test was estimated using mongrel dog blood. The PT and aPTT levels were measured through an anticoagulation test. Hence, dog blood samples were incubated with papain at specified concentrations for the evaluation. Fresh citrated dog blood was pre-incubated with various concentrations of papain for a duration of 10 min. The data shown are the mean ± SD (*n* = 3) of three independent experiments. s = Seconds.

Papain	PT (s)	aPTT (s)
Control	17.5 ± 1.8	93.9 ± 4.1
4 U/mL	18.2 ± 1.6	100.4 ± 5.8
8 U/mL	35<	400<
Normal range	14–19	75–105

## Data Availability

Data is contained within the article.
